# Enhancement of Detoxification of Petroleum Hydrocarbons and Heavy Metals in Oil-Contaminated Soil by Using Glycine-β-Cyclodextrin

**DOI:** 10.3390/ijerph16071155

**Published:** 2019-03-31

**Authors:** Wei Zhang, Yun-guo Liu, Xiao-fei Tan, Guang-ming Zeng, Ji-lai Gong, Cui Lai, Qiu-ya Niu, Yuan-qiang Tang

**Affiliations:** 1 College of Environmental Science and Engineering, Hunan University, Changsha 410082, China; zhangweisx@hnu.edu.cn (W.Z.); zgming@hnu.edu.cn (G.-m.Z.); jilaigong@hnu.edu.cn (J.-l.G.); laicui888@163.com (C.L.); niuqiuya@hnu.edu.cn (Q.-y.N.); bobtang2016@hnu.edu.cn (Y.-q.T.); 2 Key Laboratory of Environmental Biology and Pollution Control (Hunan University), Ministry of Education, Changsha 410082, China

**Keywords:** G-β-CD, petroleum hydrocarbon, heavy metal, desorption, simultaneous removal

## Abstract

Soil contamination with petroleum hydrocarbons and heavy metals is a widespread environmental problem. In recent years, cyclodextrin has attracted research interest because of its special hole structure that can form inclusion complexes with certain small molecules. However, the solubility of β-cyclodextrin (β-CD) in water is low and it crystallizes easily, leading to its low utilization in practice. In this experiment, we connected β-CD with glycine under alkaline conditions to prepare glycine-β-cyclodextrin (G-β-CD), which is water soluble, has stronger coordinating ability with heavy metals, and is more suitable for treating oil-contaminated soil. The results show that G-β-CD provides better desorption of petroleum hydrocarbons and heavy metals in soils with low organic matter content (1%) and NaNO_3_ of 0.25 mol/L at 70 g/L G-β-CD under mildly acidic (pH 5–6) conditions. The results indicate that petroleum hydrocarbons and heavy metals were removed simultaneously by means of pretreatment with G-β-CD, and the results can provide a theoretical basis for remediation of petroleum-contaminated soil.

## 1. Introduction

Oil-contamination of soil is a serious environmental problem. The total worldwide production of oil each year is approximately 2.2 billion tons, where 1.75 billion tons was produced from onshore oilfields. Approximately 8 million tons is produced during oil exploitation and lost during refining, transporting, and draining of waste oily water. This lost oil slowly leaches into the soil and groundwater [1,2]. These harmful elements will finally enter the human body through the food chain, harming human health. The main pollutants in oil-contaminated soil are petroleum hydrocarbons and heavy metals, such as Pb and Cu. Both of these metals are highly toxic and can cause cancer, abnormality, and are thus serious environmental threats. Petroleum hydrocarbons and heavy metals produce synergistic effect in soils that exacerbate the toxicity and damage to the environment, making treatment more difficult [3,4].

It is well known that oils provide a natural environment for bioremediation of polluted soils through physical, chemical, and microbiological synthesis processes [5,6]. Petroleum hydrocarbons are strongly hydrophobic and easily adsorbed in soil. These compounds do not degrade naturally by bionts in soils because of their low water solubility and biological availability. In order to enhance the desorption rate of hydrophobic organic contaminants in soils, some researchers have used surfactants or mixed organic solvent as soil desorption agents [7,8,9]. Unfortunately, organic solvents can cause secondary pollution and surfactants often form high viscosity emulsions, which are also difficult to remove from soils [10,11,12,13,14,15]. In addition, only a few studies report simultaneous removal of petroleum hydrocarbons and heavy metals from co-contaminated soils [16,17,18].

Recently, cyclodextrins have received increasing attention thanks to their peculiar hydrophobic cavity, which can form inclusion complexes with various guest molecules, thus improving the solubility of organic matter [19]. However, the solubilization action of cyclodextrin is confined to hydrophobic organic compounds with relatively low solubility. Moreover, cyclodextrin has no coordinating ability with heavy metals. Owing to these features, many applications of cyclodextrin were largely limited. Thus, modified β-cyclodextrin that coordinates with metal ions and hydrophobic organic matter was prepared. For example, some studies have shown that the formation of inclusion complexes of PAH with β-cyclodextrin (β-CD) and hydroxypropyl-β- cyclodextrin (HPCD) were investigated as a way to facilitate pollutant removal from soil [20]. Some research showed that soils polluted with phenanthrene and pyrene were almost completely depolluted by reusing methyl-β-cyclodextrin, and the loss of methyl-β-cyclodextrin in the soil was insignificant [21]. On the other hand, modified cyclodextrins with more carboxyl functional groups had stronger coordination with heavy metals. For example, carboxymethyl groups were added to the hydroxyl groups on the β-cyclodextrin core structure to form carboxymethyl-β-cyclodextrin (CMCD). This cyclodextrin derivative was found to increase the aqueous solubility of anthracene and to complex cadmium simultaneously [22,23]. Ehsana found that PCB compounds and selected heavy metals can be co-extracted efficiently from soil with three successive washes with the same washing suspension containing EDTA and cyclodextrin [24].

This study aimed to improve the water solubility of cyclodextrin and its coordination with heavy metals, and to find a more suitable material for treatment of oil-contaminated soil. Thus, glycine-β-cyclodextrin (G-β-CD), which was synthesized via the reaction of β-cyclodextrin with glycine in the presence of KOH and epichlorohydrin, can be used for simultaneous removal of organic pollutants and heavy metals from oil-contaminated soils. Simultaneous detoxification of metals and oil in the soils was investigated. This study on static desorption could provide a new method of guidance and practical application of the theory for the repair of complex pollutants.

## 2. Materials and Methods

### 2.1. Soil Characteristics and Preparation of Contaminated Soil

The soil used for the experiment was collected from a typical location in a production station at the No. 4 oil production plant of Zhongyuan Oilfield, where oil production wells were stable for more than one year. Gravel and plant root residues in the sampled soils were discarded and the soil was air-dried, sieved through a 2 mm mesh, and stored at 4 °C in darkness. Some physico-chemical characteristics of the experimental soil were measured. The soil had a pH value of 6.30, the moisture content was 1.92%, the soil respiration intensity was 0.132 mg CO_2_/g, organic matter content was 1.0%, the petroleum hydrocarbon content was 327.805 mg/kg, and the total lead and copper content were 10.792 and 9.204 mg/kg, respectively, as shown in Table 1.

### 2.2. Modification of Cyclodextrin

**Figure 1:**
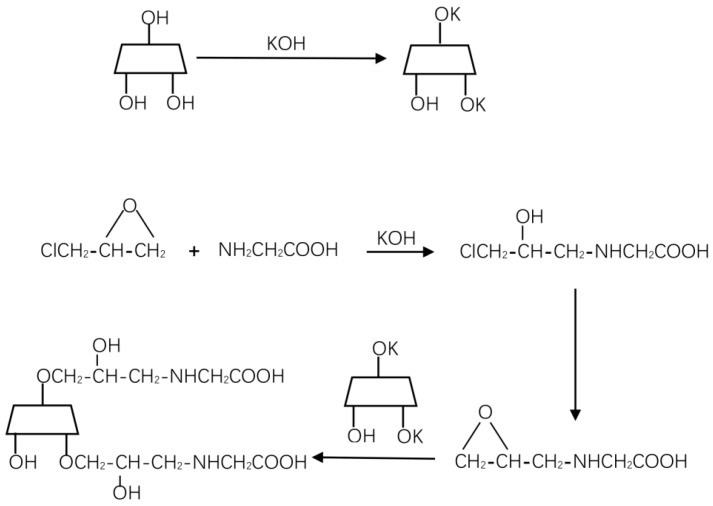
Reaction scheme for synthesis of G-β-CD.

Synthesis of glycine-β-cyclodextrin (G-β-CD) was conducted as follows. β-Cyclodextrin (β-CD) (8.100 g) was added to a 500 mL beaker, followed by the addition of distilled water (70 mL) and KOH (6.700 g) while stirring until the β-CD was thoroughly dissolved. The mixed solution in the beaker was heated to 50 °C in a water bath, with subsequent addition of glycine (7.500 g) and epoxy chloropropane (10.200 g). The mixed solution was allowed to react in a 60 °C water bath for 1 h and was subsequently allowed to cool to room temperature. Concentrated sulfuric acid was added to adjust the pH value of the mixed solution to approximately 5.5, and anhydrous ethanol (150 mL) was added and allowed to stand for filtering. The alcohol was steamed out of the filtrate on a rotary evaporator and the filtrate was concentrated to 30 mL. Then, 300 mL of methanol was added and allowed to stand overnight, filtered, and dried to yield a white glycine-cyclodextrin solid precipitate [25]. The reaction scheme for synthesis is shown in Figure 1. A few samples were collected and investigated using X-ray photoelectron spectroscopy (XPS).

### 2.3. Determination of Total Petroleum Hydrocarbon Content

Infrared spectrophotometry was used to determine the total petroleum hydrocarbon content in the soil. 1 g of petroleum-contaminated soils was added to a 50 mL centrifuge tube through a 100 mesh sieve. A soil sample extraction and detection method was performed, as described by Paiga [26]. Finally, the sample was examined with an IR petroleum determination instrument (3200 cm^−1^ to 2700 cm^−1^ scanning range).

### 2.4. Determination of Heavy Metal Content

One g of soil was added to a 50 mL centrifuge tube. Then, 25 mL of an HNO_3_-HCl solution (1:3 volume ratio) was added to the centrifuge tube. Heavy metals were hyperacoustically extracted for 25 min (40 Hz, 80 °C) and centrifuged at 4000 rpm for 10 min. The supernatant liquid was evaporated to 1 mL using a graphite digestion apparatus. The residue was cleaned for 5 min using ultrasonic agitation with 5 mL ultrapure water and subsequently centrifuged at 4000 rpm for 10 min [27]. The supernatant fluid was mixed with 1 mL of the above acid solution and diluted with water to 50 mL, and then stored at 4 °C. Atomic absorption spectrophotometry was used to determine the content of Pb and Cu.

### 2.5. Desorpion Experiment

#### 2.5.1. Solubilization of Petroleum Hydrocarbons and Heavy Metals by β-CD Before and After Modification

Ten groups of 1.00 g of contaminated soil were placed in 14 polyethylene centrifuge tubes with plugs. Thirty mL of β-CD (18 g/L) was added dropwise to seven test tubes, and 30 mL G-β-CD (18 g/L) solutions was added dropwise to the other seven test tubes. After shaking at 25 °C, samples were gathered from the tubes every hour throughout a 7 h reaction, then were centrifuged at 4000 rpm for 30 min. The concentrations of lead and copper ions in the supernatant after digestion were determined with atomic absorption spectrophotometry (AAS). The subsequent processing steps and procedure for determining the content of petroleum hydrocarbons was described by Paiga [26].

#### 2.5.2. Factors Influencing Desorption by G-β-CD

A desorption experiment was conducted using an oscillation sequencing batch test. Similarly, nine groups of 1.00 g of contaminated soil samples were placed in nine polyethylene centrifuge tubes with plugs, and 30 mL of different G-β-CD solutions (0, 10, 20, 30, 40, 50, 60, 70, and 80 g/L) solutions were added dropwise to the nine test tubes. Then, these tubes were shaken at 130 rpm at 25 °C for 6 h and centrifuged at 4000 rpm for 30 min. The concentration of petroleum hydrocarbons and heavy metals were determined as mentioned above (see Section 2.5.1).

After optimal G-β-CD desorption, the influence of acidity, ionic strength and organic content on desorption of petroleum hydrocarbons was sequentially investigated. The impact of pH on desorption of heavy metals with G-β-CD was examined by adjusting the pH of the mixture from 5.0 to 9.0. The influence of ionic strength was examined by adding NaNO_3_ (0.05, 0.10, 0.15, 0.20, 0.25, 0.30, and 0.35 mol/L), and the effect of organic matter content was examined by adding humic acid (1%, 2%, 3%, 4%, 5%, and 6% quality mark).

#### 2.5.3. Variation of Heavy Metal Speciation in Soils Before and After Desorption with G-β-CD

Four groups of 1.00 g of contaminated soil were placed in four polyethylene centrifuge tubes with plugs. Then, 30 mL of G-β-CD solutions (70 g/L) was added to the two test tubes and 30 mL ultrapure water was added to the other two test tubes. These tubes were then shaken at 130 rpm at 25 °C for 6 h. Heavy metal speciation was measured with the Tessier series extraction method [28].

#### 2.5.4. Equation of Desorption Rate

(1) $$D=\frac{C_{0}-C_{e}}{C_{0}}\times 100\text{\%}$$

where *C*_0_ and *C*_e_ (mg/L) are the initial and residual concentration of petroleum hydrocarbons and heavy metals, respectively; D represents as the rate of desorption.

## 3. Results and Discussion

### 3.1. XPS Analysis of G-β-CD

**Figure 2:**
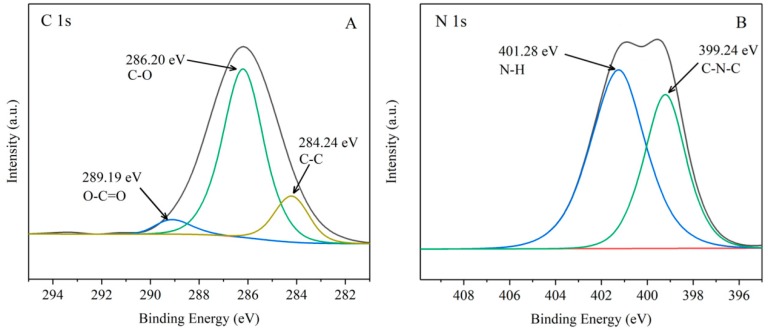
XPS spectra of G-β-CD: (**A**) C 1s and (**B**) N 1s.

The chemical composition and bonding configuration of β-CD and G-β-CD were investigated using XPS. The XPS survey scan contains C 1s and O 1s peaks in β-CD and G-β-CD. The intensity of the C 1s and O 1s peaks in G-β-CD was stronger than those in β-CD. N 1s peaks were also observed in G-β-CD. As shown in Figure 2A, high resolution XPS spectra of C 1s in G-β-CD can be deconvoluted into three peaks located at 284.20, 286.2, and 289.19 eV, which are assigned to the sp2 C-C, C-O (hydroxyl), and O-C=O (carboxylate carbon) bonds, respectively. The high resolution spectrum of N 1 s (Figure 2B) was deconvoluted into two peaks located at 399.24 and 401.28 eV, which are attributed to C-N-C and N-H bonds. The XPS analysis further confirmed that G-β-CD contained amino and carboxyl groups, which suggests that glycine can be used to modify β-CD.

### 3.2. Effect of β-CD and G-β-CD on Solubilization of Petroleum Hydrocarbons and Heavy Metals

**Figure 3:**
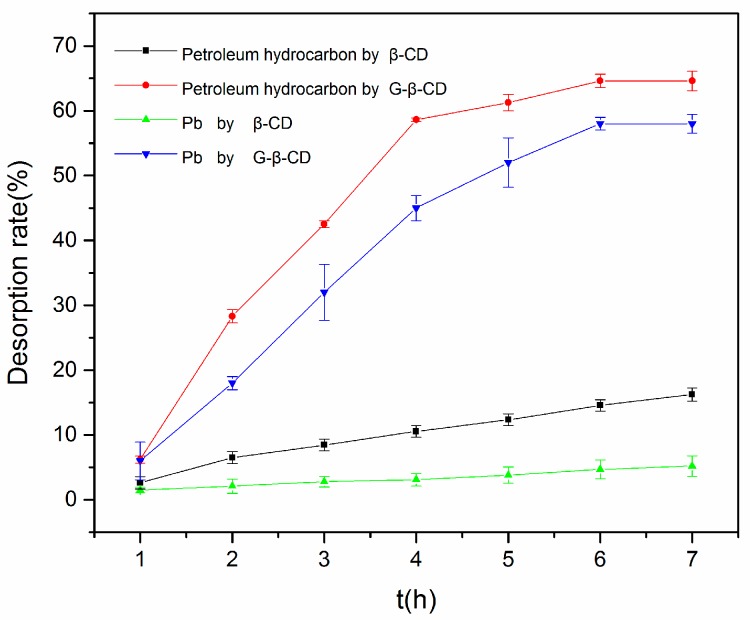
Desorption rate of petroleum hydrocarbons and lead with β-CD and G-β-CD.

The effects of β-CD and G-β-CD on solubilization of petroleum hydrocarbons and heavy metals are shown in Figure 3, where the effect of G-β-CD on solubilization of petroleum hydrocarbons and heavy metals is about 55% and 48%, respectively, which is far stronger than the desorption rate provided by β-CD (12% and 8%, respectively). One of the main reasons for this is that G-β-CD is much more soluble than β-CD. In addition, the effect of G-β-CD on the solubilization capacity of petroleum hydrocarbons has increased significantly compared to β-CD. Moreover, coordination interactions between metal cations and amino and carboxyl groups increased markedly, which could significantly increase the heavy metal desorption rate. From this figure, we can see that petroleum hydrocarbons and heavy metals can reach desorption equilibrium after 6 h; thus, 6 h was taken as the desorption balance time in the following procedures.

### 3.3. Factors Affecting Desorption

#### 3.3.1. Effect of Initial Concentration of G-β-CD on Desorption

**Figure 4:**
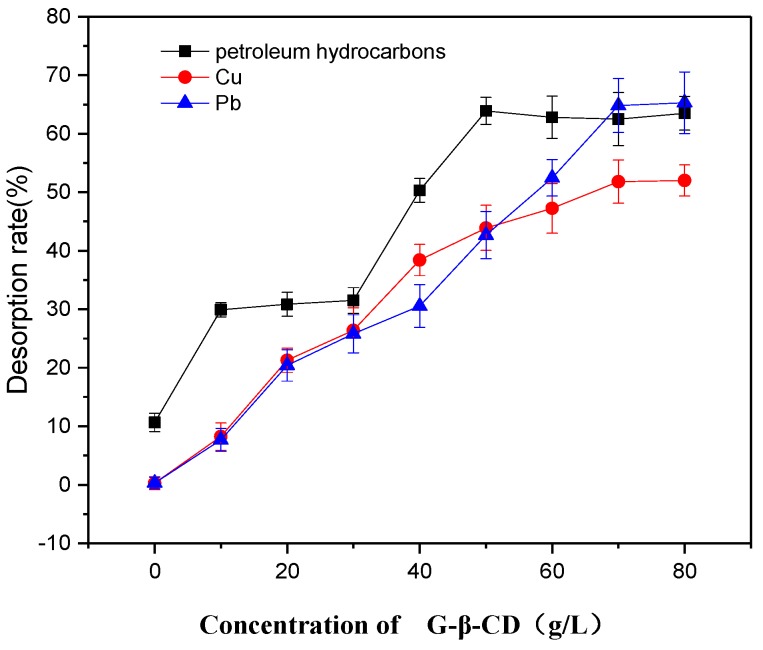
Effect of the initial G-β-CD concentration on desorption.

Desorption of petroleum hydrocarbons and heavy metals with at varying initial concentration of G-β-CD was also investigated. As shown in Figure 4, the solubilization rate of petroleum hydrocarbons increased as the initial concentration of G-β-CD increased. This was primarily due to the higher solubilization capacity of G-β-CD at higher concentration. Petroleum hydrocarbons gradually transfer from the surface of the soil to the liquid phase by redistribution between solid and liquid phases, which resulting in higher desorption. Meanwhile, desorption of Pb and Cu increased as the initial concentration of G-β-CD increased. The higher concentration of G-β-CD may reduce coordination of metal ions to a greater extent; thus, heavy metal ions initially adsorbed on the surface of the soil will gradually move to the liquid phase by redistribution between solid and liquid phases, thereby increasing the desorption capacity of heavy metals in soil [29]. Figure 4 shows that petroleum hydrocarbons, Pb, and Cu have high desorption rate at an initial G-β-CD concentration of about 70 g/L; thus, 70 g/L can be taken as the optimal cyclodextrin concentration.

#### 3.3.2. Effect of pH on Desorption

**Figure 5:**
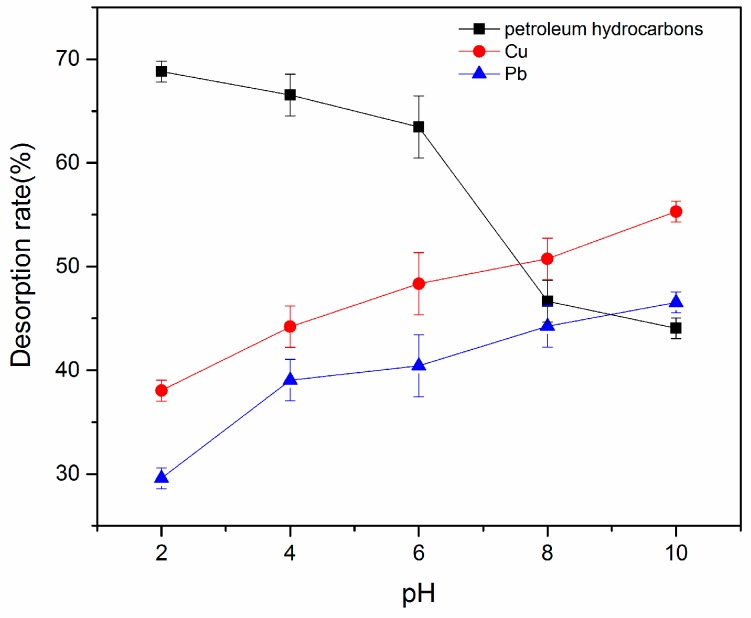
Effect of pH on desorption.

The solution pH is one of the most vital parameters to consider when optimizing the desorption process. The effect of pH on desorption of petroleum hydrocarbons and heavy metals with G-β-CD was investigated. As shown in Figure 5, the petroleum hydrocarbon desorption rate gradually decreased as pH increased. G-β-CD is positively charged in acidic conditions due to the relatively easy protonation of amine groups, while soil contains many positively charged ions in acidic conditions. The absorption rate begins to saturate at low pH due to electrostatic interaction between G-β-CD and positively charged ions, resulting higher desorption of petroleum hydrocarbons in acidic conditions. Electrostatic repulsion gradually weakened as the pH increased, leading to lower desorption. Moreover, it was found that the structure of G-β-CD was destroyed in strongly alkaline conditions, and its encapsulation ratio tended to decline, thus decreasing the desorption rate.

The desorption rate of Pb^2+^ and Cu^2+^ also gradually increased as the pH increased. G-β-CD carries various surface functional groups (primarily oxygen-containing groups, e.g., -COOH; and -OH). Metal ions exhibit less competition with protons for binding sites, and more binding sites are released due to deprotonation of functional groups at higher pH [30]. This may arise if amino in G-β-CD combined with hydronium ions repels metal ions. H^+^ combines with OH^-^ in alkaline conditions [31]. Therefore, carboxyl in G-β-CD carries net negative charge and easily binds metal ions. All of these factors increase the desorption rate of metal ions at higher pH.

#### 3.3.3. Effect of Ionic Strength on Desorption

**Figure 6:**
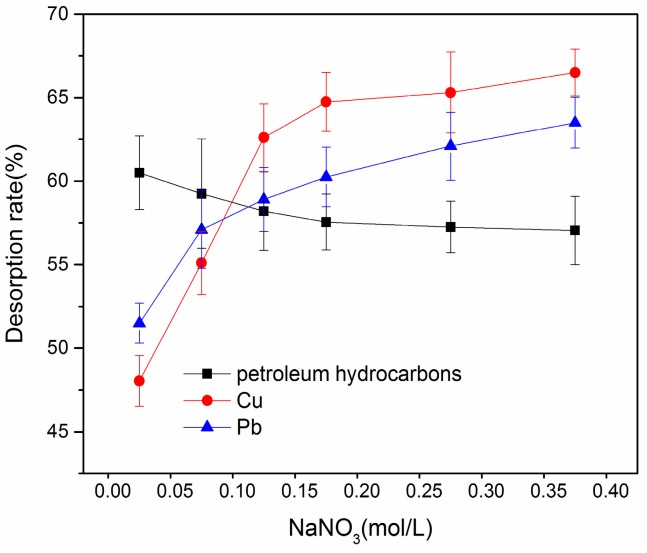
Effect of ionic strength on desorption.

The data in the Figure 6 shows that the desorption rate of petroleum hydrocarbons decreased as the increase of ionic strength decreased, which may arise because the solubility of organic matter in nonpolar or weakly polar water usually decreases as the ionic strength increases (i.e., salting out effect). Lower solubility decreased the rate of petroleum hydrocarbon desorption in soil. Turner proposed that the increased ionic strength can compress the soil surface, similar to an electric double layer, thus changing the structure of organic matter and increasing desorption of petroleum hydrocarbons [32]. Therefore, the increased ionic strength also compresses the surface of clay and organic matter in soil on the surface of the electric double layer structure, which decreased net negative charge in soil, even when the soil is close to its isoelectric point. This is more conducive to adsorption of hydrophobic organic compounds, resulting in the decreased desorption rate of petroleum hydrocarbons.

The desorption rate of Pb and Cu increased as the increase of ionic strength, which is primarily due to the presence of K^+^, which can compete with metal ions adsorption sites in soil. Furthermore, potassium nitrate produced by electrolysis of PbNO_3_ from adsorption sites can generate soluble salts that dissolve in solution. In addition, increasing the ionic strength will also strengthen the coordination effect of functional group on G-β-CD, which increases the desorption capacity [29]. From this figure, the desorption rates of petroleum hydrocarbons and heavy metals was relatively high at an NaNO_3_ concentration of approximately 0.25 mol/L.

#### 3.3.4. Effect of Organic Matter Concentration on Desorption

**Figure 7:**
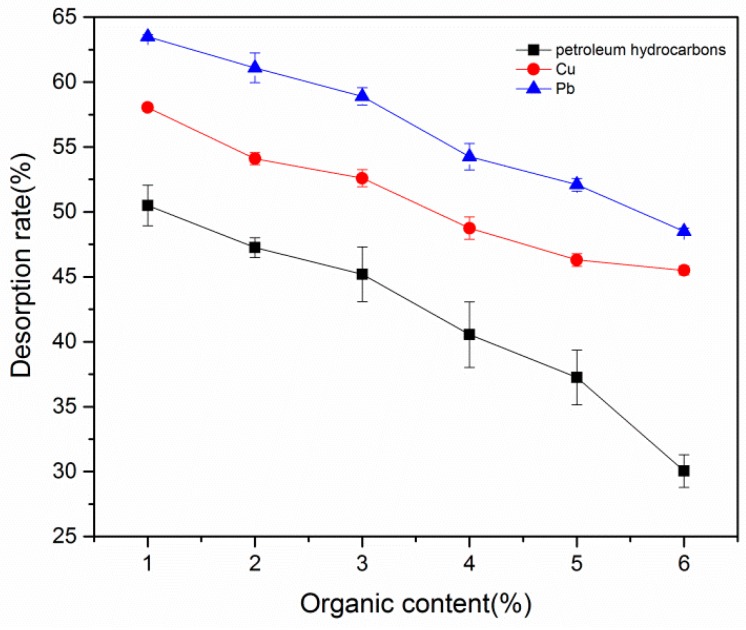
Effect of organic matter concentration on desorption.

The influence of the mass fraction of humic acid on petroleum hydrocarbon degradation is shown in Figure 7. The desorption rate of petroleum hydrocarbons gradually decreased as the content of humified organic matter in soil increased, which may arise due to the increased degree of polymerization and a decreased number of oxygen containing functional groups in organic matter. The desorption rate of petroleum hydrocarbons, which are hydrophobic organic pollutants, was slow during oxidation of organic matter; thus, the desorption rate of petroleum hydrocarbons gradually decreased. Meanwhile, the desorption rate of G-β-CD for Pb and Cu also gradually decreased as the concentration of organic matter in soil increased because the organic matter contained more oxygen-containing functional groups, e.g., carboxyl, phenol, and hydroxyl. These functional groups have very strong affinity for heavy metals and organic matter, which strengthened coordination and decreased the desorption rate of heavy metals in soil.

#### 3.3.5. Speciation of Metal Ions in Oil-Contaminated Soil Before and After G-β-CD Desorption

**Figure 8:**
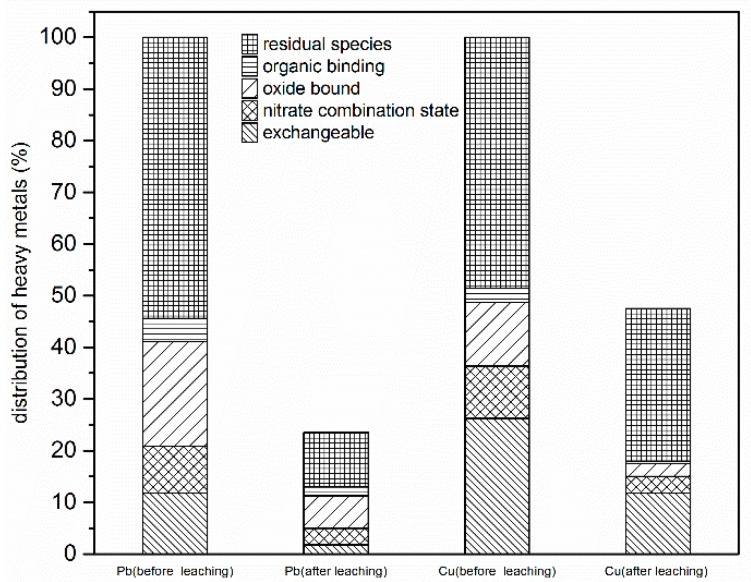
Speciation of metal ions on oil-contaminated soil before and after G-β-CD desorption.

The data in Figure 8 show changes in heavy metal speciation. After desorption, the water soluble, exchangeable Pb concentration decreased to 70%. The amount of carbonate bound to Pb was less than the detection limit. The concentration of residual Pb decreased to 8.28%. Carbonate-bound Cu, iron-manganese oxide bound Cu, and residual Cu were high before desorption. The concentration of Cu residue did not change largely due to desorption, but the other two forms of Cu decreased by 60%. In general, the exchangeable form of heavy metal in soil is the fraction that can be easily adsorbed. In addition, carbonate-bound Cu was converted to a water soluble form as the pH changed; thus, carbonate-bound Cu could be easily converted to the aqueous phase and absorbed by organisms [33]. After being desorbed with G-β-CD, the concentration of exchangeable and carbonate-bound Cu decreased sharply. In particular, Pb has a strong retention capacity in soil; hence, most residual Pb was transferred to an elution solution, resulting in the decreased bioavailability and toxicity of heavy metals [34].

## 4. Conclusions

In view of these results, one can conclude that G-β-CD could simultaneously increase the apparent aqueous solubility of petroleum hydrocarbons and complex with heavy metals. The solubilization effect on petroleum hydrocarbons significantly increased as the concentration of G-β-CD increased. When the G-β-CD concentration was 70 g/L, the desorption rate of petroleum hydrocarbons and heavy metals (Pb and Cu) in mildly acidic conditions of pH 5–6 reached 63.90% in soil with 0.25 mol/L NaNO_3_ and low organic matter content (1%). G-β-CD provided stronger desorption of petroleum hydrocarbons and heavy metals in soils, compared with β-CD. Analysis of the variation of heavy metal speciation before and after eluting with G-β-CD revealed that, soluble species as well as organic bound and carbonated fractions of Pb and Cu adsorbed in soil were washed away, dropping below the detection limit. The content of oxides and debris decreased slightly. Accordingly, the toxicity and biological availability of heavy metal in oil-polluted soils decreased.

The results show that the addition of G-β-CD elution had significant removal effects on the petroleum hydrocarbons and heavy metal co-contaminated soil. This system can be used to treat oil-contaminated soil and subsequently remove residual liquids. Further studies on microbial responses to cyclodextrin derivatives during biodegradation of organic matter should be conducted. G-β-CD should be considered as a potential candidate for treating oil-contaminated soil due to its low cost and the low environmental impact of cyclodextrins.

## Figures and Tables

**Table 1:** Characteristics of the studied soil.

pH	Moisture Content (%)	Soil Respiration Intensity (mg CO_2_/g)	Organic Matter Content (%)	Petroleum Hydrocarbon Content (mg/kg)	Total Lead Content (mg/kg)	Total Copper Content (mg/kg)
6.30	1.92%	0.132	1.0	327.805	10.792	9.204
